# Unsupervised detection of high-frequency oscillations in intracranial electroencephalogram: promoting a valuable automated diagnostic tool for epilepsy

**DOI:** 10.3389/fneur.2025.1455613

**Published:** 2025-03-26

**Authors:** Wenjing Chen, Tongzhou Kang, Md Belal Bin Heyat, Jamal E. Fatima, Yuanning Xu, Dakun Lai

**Affiliations:** ^1^West China Hospital, Sichuan University, Chengdu, China; ^2^Biomedical Imaging and Electrophysiology Laboratory, School of Electronic Science and Engineering, University of Electronic Science and Technology of China, Chengdu, China; ^3^CenBRAIN Neurotech Center of Excellence, School of Engineering, Westlake University, Hangzhou, China

**Keywords:** medical machine learning, auto-encoder, epilepsy, EEG signal, AI for surgery, wavelet transform, unsupervised learning

## Abstract

**Objective:**

This study aims to develop an unsupervised automated method for detecting high-frequency oscillations (HFOs) in intracranial electroencephalogram (iEEG) signals, addressing the limitations of manual detection processes.

**Method:**

The proposed method utilizes an unsupervised convolutional variational autoencoder (CVAE) model in conjunction with the short-term energy method (STE) to analyze two-dimensional time-frequency representations of iEEG signals. Candidate HFOs are identified using STE and transformed into time-frequency maps using the continuous wavelet transform (CWT). The CVAE model is trained for dimensionality reduction and feature reconstruction, followed by clustering of the reconstructed maps using the K-means algorithm for automated HFOs detection.

**Results:**

Evaluation of the proposed unsupervised method on clinical iEEG data demonstrates its superior performance compared to traditional supervised models. The automated approach achieves an accuracy of 93.02%, sensitivity of 94.48%, and specificity of 92.06%, highlighting its efficacy in detecting HFOs with high accuracy.

**Conclusion:**

The unsupervised automated method developed in this study offers a reliable and efficient solution for detecting HFOs in iEEG signals, overcoming the limitations of manual detection processes of traditional supervised models. By providing clinicians with a clinically useful diagnostic tool, this approach holds promise for enhancing surgical resection planning in epilepsy patients and improving patient outcomes.

## 1 Introduction

Epilepsy, a complex neurological disorder characterized by recurrent and unpredictable seizures, affects over 65 million people globally, constituting a significant public health burden (1). Despite considerable advancements in antiseizure medications and other therapeutic modalities, a substantial proportion of individuals with epilepsy continue to experience uncontrolled seizures, severely impacting their quality of life and daily functioning (2, 3). For individuals with medically refractory epilepsy, surgical intervention remains a viable option aimed at achieving seizure freedom and improving overall well-being. Central to the success of epilepsy surgery is the accurate localization and delineation of the epileptogenic zone (EZ), the area of the brain responsible for initiating and propagating seizures. Accurate delineation of the EZ is critical to ensure maximal resection of epileptogenic tissue while minimizing the risk of postoperative neurological deficits.

Intracranial electroencephalogram (iEEG) recordings play a pivotal role in the pre-surgical evaluation of epilepsy patients (4) by providing direct and high-temporal-resolution insights into the spatial and temporal dynamics of epileptiform activity within the brain. Over recent years, high-frequency oscillations (HFOs) in iEEG signals have emerged as potential biomarkers for the epileptogenic tissue, offering valuable insights into the pathophysiology of epilepsy and guiding surgical decision-making. However, the manual detection and characterization of HFOs present significant challenges. Traditional methods rely on labor-intensive and subjective processes for feature extraction and annotation, leading to variability in results and hindering scalability for large-scale analysis. Moreover, the manual analysis of iEEG recordings is time-consuming and resource-intensive, limiting its clinical utility in routine practice. Most recently, Zhang et al. (4) explored whether the unique mechanisms of pathological and physiological HFOs are reflected in their signal morphology within intracranial EEG (iEEG) recordings. They also examined whether this mechanism-based distinction could be replicated using a deep generative model.

In response to these challenges, various HFOs detection models based on semi-and unsupervised machine learning attracted researchers’ attention, such as the K-means model (K-means) (5), the Gaussian mixture model (GMM) (6), the fuzzy c-means model (FCM) (7), and mean-shift algorithm (7). Blanco et al. (8) reported a K-means model to automatically detect HFOs, which screened out all possible HFOs (pHFOs) with the RMS method first, then extracted 7 features of HFOs signals, and used a K-means model to cluster them. Alternatively, Liu et al. (5) screened the pHFOs signal by calculating the Hilbert transform envelope of the original EEG signal and then used the K-means model to cluster them with three features of the sub-band power ratio, high band entropy, and peak-sink energy ratio. Moreover, Liu et al. (6) used the root mean square method to screen pHFOs, performed time-frequency analysis to extract features and finally adopted the GMM for clustering them. Wu et al. (7) used the root mean square method to screen pHFOs from EEG data and extracted four features: short-term energy, fuzzy entropy, power ratio, and spectral centroid. He proposed the quadric error metrics (QEM) algorithm to calculate the optimal number of clusters, and finally, real HFOs were detected automatically by FCM clustering with the number of cluster centers being. Du et al. (9) proposed a semi-supervised learning-based method for HFOs detection in EEG signals, which first used wavelet entropy and the Teanger energy operator to detect pHFOs and then used labeled EEG data to initialize the K-means model. Migliorelli et al. (10) proposed a HFOs detection algorithm based on the S-transform and the GMM algorithm. The algorithm performed Stockwell transformation on EEG signals to extract features and then used GMM to perform cluster analysis on the features. Wan et al. (11) extracted four features by combining a variety of signal analysis methods to analyze the pHFOs signals, such as the Stockwell forward and inverse transformation, the singular value decomposition, etc., and clustered them with the improved FCM algorithm. However, considerable difficulties of such supervised learning and semi-and unsupervised methods seem to be built in, such as exhausting and subjective feature extraction from either the time domain or the frequency domain, feature selection by ranking them with statistical tests. Although recently deep learning models for HFOs detection without any issues as above, time-consuming tasks for manually labeling pHFOs events to train supervised deep learning model is still ineluctable, as reported in (12) and one of our previous work (13).

We develop an unsupervised method for automatically detecting and characterizing HFOs in iEEG signals. Leveraging advancements in machine learning and signal processing, our approach aims to streamline the identification of HFOs while ensuring accuracy and reproducibility. Specifically, we propose a novel framework that integrates a convolutional variational auto encoder (CVAE) model with the short-term energy method (STE) to analyze two-dimensional time-frequency representations of iEEG signals. Through unsupervised feature extraction and clustering techniques, our method seeks to enhance the efficiency and objectivity of HFOs detection, facilitating precise localization of the EZ and guiding surgical resection planning. By evaluating the performance of our automated approach on a diverse dataset of clinical iEEG recordings from patients with medically intractable epilepsy, we aim to demonstrate its potential as a valuable tool for neurosurgeons and epileptologists in the management of epilepsy.

## 2 Materials and methods

**Figure 1:**
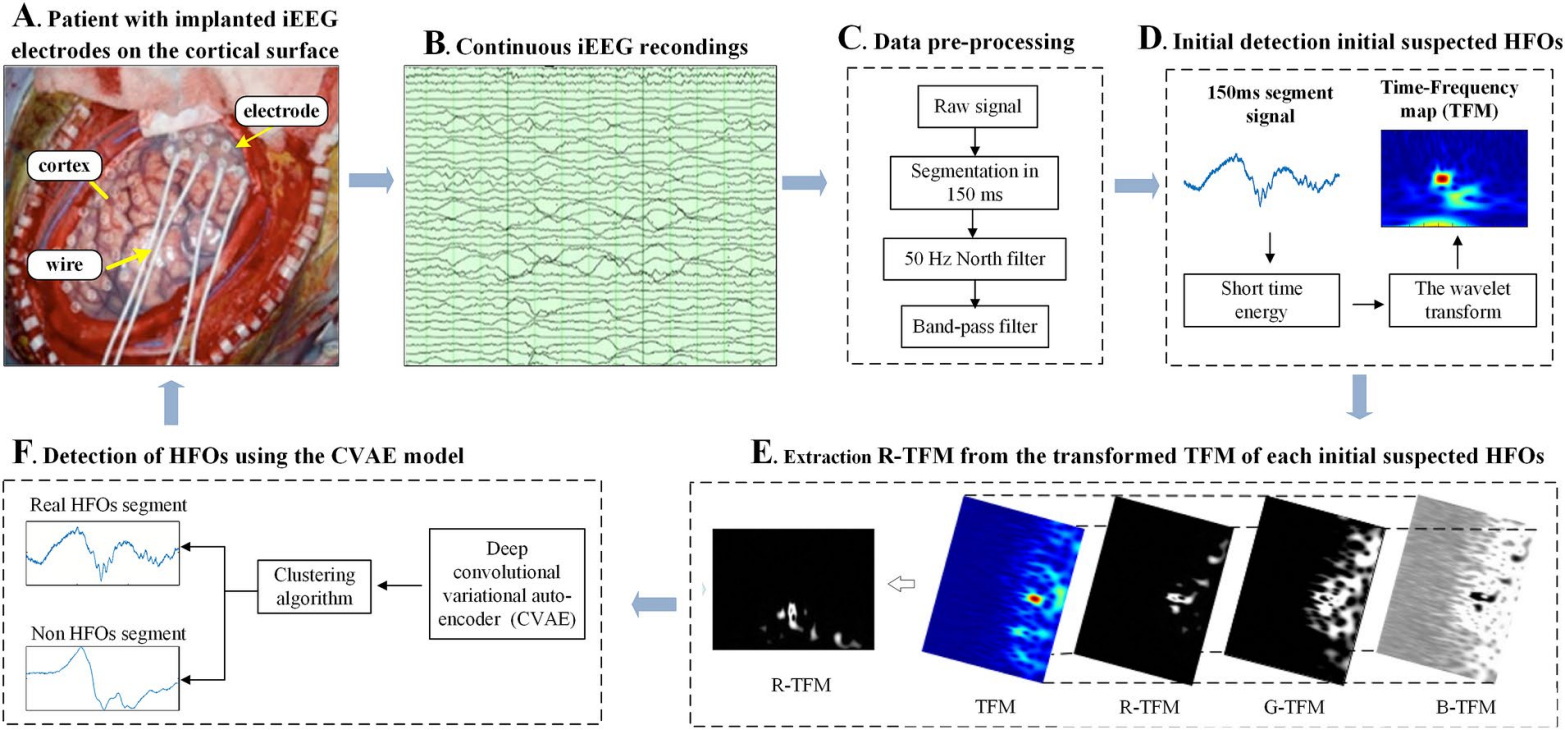
Schematic diagram of the proposed HFOs detector using the convolutional variational automatic encoder (CVAE) model **(F)** fed by a two-dimensional time-frequency map **(E)** of intracranial electroencephalogram (iEEG) signals **(B)**, which were recorded by intracranial EEG electrodes **(A)** and subsequently pre-processed by digital filters **(C)**, noted that the short time energy of each segment was calculated for an initial detection of suspected HFOs **(D)**.

The proposed automatic HFOs detector in terms of its 2D time-frequency map, which is based on the CAVE model together with the STE method 2D CNN (Figure 1).

### 2.1 Clinical dataset

Five consecutive patients with medically intractable epilepsy were involved in the study. For each patient, pre-surgical monitoring was performed in the epilepsy monitoring unit of the Department of Neurosurgery, West China Hospital, Chengdu, Sichuan Province, China. this hospital. And, the classification of postoperative outcomes for epilepsy surgery of each patient was given according to Engel’s score, including Class I: free from disabling seizures; Class II: Rare disabling seizures (almost seizure free), Class III: worthwhile improvement, Class IV: No worthwhile improvement. Follow-up of all patients was >1 year.

Specifically, around 46–110 intracranial EEG electrodes formatted in subdural silastic grids were implanted on the cortical surface of every patient for a continuous EEG recording, as shown in Table 1. All raw iEEG data included in this study conformed to the same characteristics: the continuous duration of recordings was greater than 2 h in the awake state, the sampling rate was 4,096 Hz, the data were recorded by using a subdural silastic grid with 46–110 electrodes in 4 mm diameter electrode contracts and 10 mm inter-electrode spacing with XLTEK EMU128FS system (Natus Neurology, USA), and no hardware filters were used during data acquisition (13). Five patients with refractory epilepsy were screened according to the criteria and recorded for a total of 14 h. Note that not all electrode channels are available, where totally corrupted channels with artifacts and noise enormous amplitudes due to improper patient movement were excluded. All patients signed the consent form, and the ethics committee [2022-IRB Review (9)] on biomedical research at West China Hospital of Sichuan University, China, approved the proposed study.

**Table 1:** Intracranial EEG recording in patients with medically intractable epilepsy.

Patient No., Gender	Patient Age (year)	Pathology Diagnosis	Implantation Sites	No. of Selected/Total Channels (n)	Recording Length in Awake State (hrs)	Engel Class (year)
Pt1, Male	26	FCD	LTOL, RFTPL	66 (84)	4	II (2)
Pt2, Female	19	DG	LFTPL	54 (74)	3	III(1.4)
Pt3, Male	20	No excision	RTP	90 (110)	3	IV
Pt4, Male	23	HS	LATL	24 (84)	2	I(2)
Pt5, Female	54	LGG	BTL	38 (46)	2	IV(1)

FCD, Focal Cortical Dysplasia; DG, Dense Gliosis; HS, Hippocampal Sclerosis; LGG, Low Grade Glioma; LTOL, Left Temporal Occipital Lobe; RFTPL, Right Frontal Temporal Parietal Lobe; LFTPL, Left Frontal Temporal Parietal Lobe; RTP, Right Temporoparietal Pillow; LATL, Left Anterior Temporal Lobe; BTL, Bilateral Temporal Lobe.

### 2.2 Data pre-processing and initial detection of suspected HFOs

In this study, to adapt to data filtering and pre-screening HFOs, all collected iEEG recordings were firstly down-sampled to 2,560 Hz. Then, each iEEG recording war is cut into segments with a length of 150 ms for further data preprocessing. Moreover, each segment was pre-processed by a 50 Hz multi-notch filter and a 4^th^ order Butterworth band pass filter (80–500 Hz) so as to remove power frequency interference and other electrophysiological signal interference in EEG signals, respectively. Subsequently, the short time energy (STE) per frame was calculated from the filtered iEEG data using a 10 ms sliding window (14). The mathematical expression of the STE are mentioned in Equation 1:


(1) $$E^{*}\left(t\right)\;=\;\frac{1}{N}\overset{t}{\underset{k=t-N+1}{\sum }}x^{2}\left(k\right)$$

Where, E*(t) is the short-term energy of the frame, starting at t. x(k) is the kth point of pHFO, and N is the number of sample points contained in the frame. STE defines a threshold that exceeds 3 times the standard deviation (SD) of the mean of the STE signal. The 150 ms raw iEEG signal segment was identified as pHFO when the STE values for three consecutive frames all exceeded the defined threshold. The center of pHFOs is located in the middle of these three adjacent segments. The STE estimation threshold E0 is mentioned in Equation 2 below:


(2) $$E_{0}=E_{av}+k^{*}SD$$

Where. k is the weight of SD, and Eav is the average of STE. The parameter k needs to be tuned to strike a balance between the higher sensitivity of the STE estimator and fewer falsely detected HFOs events.

In this work, the method of STE was designed as an initial detection of HFOs for rapidly selecting all candidate HFOs with lower cost of computation in raw iEEG in this study, as we know that it would take lot of time for the subsequent CNN classifier if all of raw iEEG data are directly input in terms of 2D time-frequency map. So, the value of the parameter k is set for an initial detector with a high sensitivity and a low specificity so as to find all suspicious HFOs or 100% of the HFOs with respect to the gold standard data. In this work, the k value was specifically set as 5, and a total of 4,042 fragments are obtained by applying STE estimation.

### 2.3 Feature extraction based on time-frequency analysis

Feature extraction and continuous wavelet transform (CWT) are the main time-frequency analysis methods used in previous studies of HFOs (15). Feature extraction in the time and frequency domains reduces 1D iEEG signals to feature scalars, while CWT delineates 1D iEEG signals on a 2D TFM, as shown.

**Figure 2:**
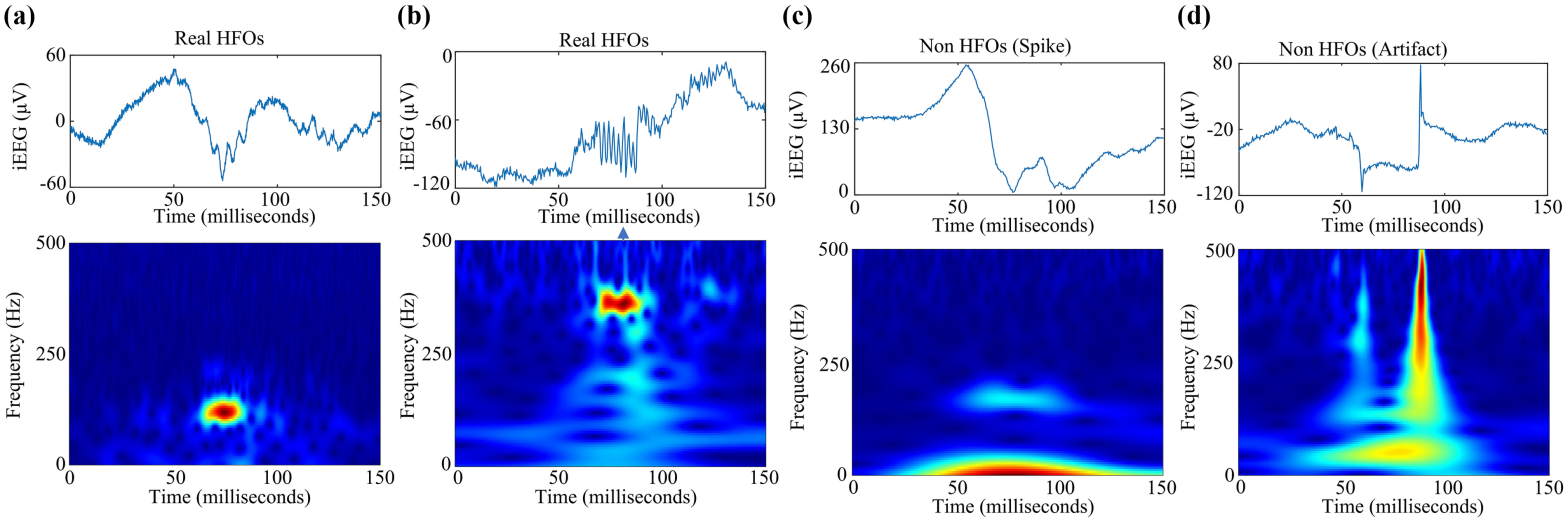
Examples of the time-frequency maps of pHFOs groups including **(a)** real HFOs segments, **(b)** non HFOs segments **(c)** spike, and **(d)** artifact.

To preserve the complete information contained in pHFO events, we employ the analytic Morse CWT function to generate TFM (16). Compared with other commonly used time-frequency representations, such as Morlet wavelets and derivatives of Gaussian wavelets, analytical Morse wavelets are more suitable for describing specific time and frequency components (17). In this study, each TFM is formatted as a color map with the size of 875 × 656, which is a calculated energy scale plot that highlights the dominant frequency components on the time scale in red. Through in-depth observation, we found that pHFOs can be divided into real HFOs segments and non HFOs segments such as spikes and artifacts. Figure 2 shows examples of the obtained TFM pHFOs.

Moreover, this study employed the gold standard for the detection of HFOs (18). And two neuro-electrophysiologists recognized each suspected HFO in terms of TFM accordingly as a real HFO or a non-HFO with the help of our custom designed GUI software on the basis of MATLAB software package (Mathworks, Natick, MA), as the 2D time-frequency map has demonstrated to be a promising approach to distinguish valid HFOs among background (6). Specifically, real HFOs event is represented obviously by an isolated island phenomenon in its time-frequency map, while such island phenomenon cannot be found for false HFOs such as spike and artifact, as shown in Figures 2a–d. Therefore, each TFM could be marked visually as HFOs or not clearly when the doctor inspected iEEG data.

It should be noted that all labeled TFMs were used only to evaluate the proposed method’s performance and train other existing supervised detectors in our comparative experiments. Finally, a total of 4,042 TFMs were recognized visually as 1,611 real HFOs and 2,431 non-HFOs (including 1,627 spikes and 804 noises). Importantly, each visual recognition of HFOs in terms of TFM not only depends on morphological differences between pHFOs but also utilizes the dominant frequency components colored by red pixels. Since the TFM of HFOs presents red islands, it is easy to distinguish Rs and FRs from erroneously detected HFOs. Therefore, The TFM is decomposed into three grayscale images representing the red, green, and blue channels of the TFM in this study. Furthermore, we extracted the red channel of TFM and considered it as a surrogate for TFM, named R-TFM in the work. Note that this intuitively simple but fairly efficient process serves several purposes, including reducing dimensionality and improving detection performance.

### 2.4 CVAE-based HFOs detector

**Figure 3:**
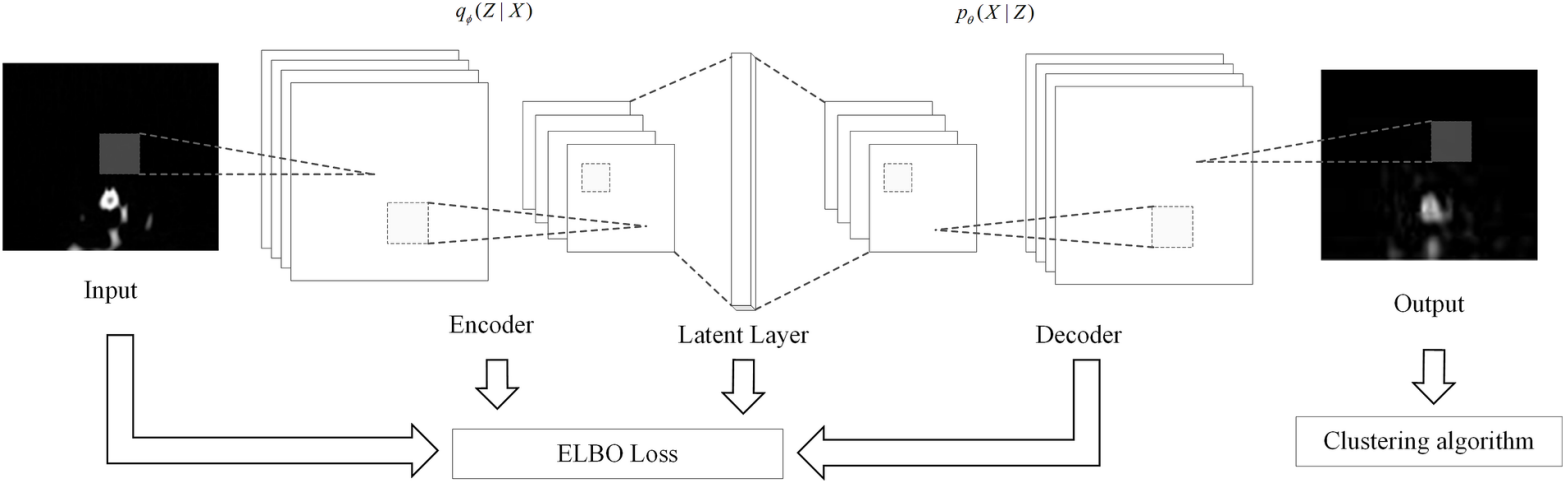
The proposed convolutional variational auto-encoder model framework.

CVAE is an improvement over traditional auto encoders. It uses convolutional and pooling layers instead of the original fully connected layers, which are more efficient for images (19, 20). The proposed CVAE detector in this work is shown in Figure 3. We trained CVAE to remove noise in R-TFM and extract high-level features. Supposed X = (X_ij_, i < m and j < n), The method uses an encoder to obtain the overall posterior inference of the R-TFM. The encoder contains two convolutional layers with kernel sizes (m × n) of 32 × 32 and 16 × 16. We introduce q*Φ*(Z|X) to denote the encoder, where Φ represents the parameters of the encoder. N stands for Gaussian distribution. To address the problems caused by sampling operations in CVAE training, we employ the reparameterization trick proposed by Kingma et al. (18), Rezende et al. (21), and mentioned in Equation 3 below:


(3) $$\begin{array}{l} \left(\mu ,\;\log\left(\sigma \right)\right)\;=\;\mathrm{Encoder}\left(X\right)\\ q_{\Phi }\left(Z|X\right)\;=\;N\left(Z;\;\mu ;\;\mathrm{diag}\left(\sigma \right)\right) \end{array}$$

The decoder is just a mirror encoder, which uses two convolutional transpose layers to define the conditional distribution of X as p*θ* (X|Z), where θ denotes the parameters of the decoder. Model the latent distribution prior p (z) as a unit Gaussian distribution. The network takes Z = [*z_1_, z_2_, …. z_l_*] as input, outputs a set of parameters pθ (X|Z), and then uses its output to reconstruct the denoised X. Variational auto encoders are usually trained by maximizing the evidence lower bound (ELBO). To improve the potential compactness of ELBO, CVAE is trained by minimizing the ELBO loss, which is defined as (21) and Equation 4 below:


(4) $$L^{\mathrm{CVAE}}\left(\theta ,\;\phi \right)\;=\;E_{q\left(x\right)}\{E_{q_{\phi }\left(z|x\right)}[\log(\frac{p_{\theta }\left(x,\;z\right)}{q_{\phi }\left(z|x\right)}]\}$$

Since the classification mechanism of R-TFM is relatively simple, easy to implement, and has a fast convergence speed, the K-means algorithm is used to classify it. Specifically, the reconstructed R-TFM is expanded into the array by vectorising the pixel matrix in a column-wise style (22). The K-means algorithm takes a vectorized version of the reconstructed R-TFM (size 64 × 64) as input and divides the reconstructed R-TFM into four groups (K = 4).

To assign these four sets of reconstructed R-TFMs to specific pHFOs classes (R, FR, spikes, and artifacts), we employed an HFOs distinguishing feature called the spectral centroid (SC), which is in the HFOs class Medium larger (8). SC denotes the frequency corresponding to the spectral centroid of the input data, which in our work are pHFOs (Equation 5):


(5) $$SC\;=\;\frac{\overset{N/2}{\underset{k\;=\;0\;}{\sum }}\frac{k}{NT}{\left|M\left[k\right]\right|}^{2}}{\overset{N/2}{\underset{k\;=\;0}{\sum }}{\left|\;M\left[k\right]\right|}^{2}}$$

Where, T is the sampling period, and *N* is the number of sampling points in the pHFOs. M[k] is the multiuser power spectral density estimate, mentioned in Equation 6 below:


(6) $$M\left[k\right]\;=\;\overset{N-1}{\underset{n\;=\;0}{\sum }}w\left[n\right]x\left[n\right]e^{-j\left(2\pi /N\right)nk},\;k\;=\;0,1,2\ldots ,N$$

Where, w[n] and x[n] are Hamming windows and pHFOs, respectively. For each cluster of reconstructed R-TFM, its SCs were calculated with its corresponding pHFOs.

### 2.5 Evaluation of the proposed CAVE detector

The ground-truth labels of the TFM dataset are used to evaluate the performance of the CVAE based HFOs detector using a fivefold cross-validation. True positives (TP), true negatives (TN), false positives (FP), and false negatives (FN) are evaluated by comparing predicted labels with ground truth labels. TP represents the number of pHFOs correctly classified as HFOs (Rs and FRs), and TN represents the number of pHFOs correctly classified as falsely detected HFOs (spikes and artifacts). The mathematical expression of the evaluation methods (23, 24) are mentioned in Equations 7–9:


(7) $$\mathrm{Accuracy}=\;\frac{TP\;+\;TN}{TP\;+\;TN\;+FP\;+FN}$$

(8) $$\mathrm{Sensitivity}\;=\;\frac{TP\;}{TP\;+\;FN}$$

(9) $$\mathrm{Specificity}\;=\;\frac{TN}{TN\;+FP}$$

## 3 Results

Our study’s main steps are preprocessing, initial detection of suspected HFOs, feature extraction, and algorithm implementation. To verify the effect of the deep unsupervised model proposed in this study, we used two supervised algorithms including K-nearest neighbor (KNN) (25) and support vector machine (SVM) (26, 27) to classify R-TFM and then used four unsupervised algorithms currently commonly used in HFOs automatic detection to cluster R-TFM (size 64 × 64) analysis and comparison. Specifically, these unsupervised algorithms include the mean-shift algorithm, K-means algorithm, GMM algorithm, and FCM algorithm. At the same time, we compared the clustering performance of the model without CVAE reconstruction and after CVAE reconstruction to verify the reconstruction effect on the same clinical dataset as mentioned above. For the proposed model of CVAE, trainings with various parameters are a time-consuming process. However, the training process can be carried out off-line. In this study, the proposed unsupervised CVAE by using 2D TFMs as input can be trained in less than 20 min. As for the testing process, several experiments as mentioned above were performed on the recorded 14 h clinic iEEG data, and it was revealed approximately that the testing process is about 1 ms for one sample of 150 ms data.

In this study, the designed CAVE model ran on the deep learning framework Tensorflow 2.2 in the Microsoft Windows 10 operating system. All experiments including model training and test were conducted on a desktop computer with an Intel i9-10900K with 32 GB memory and an NVIDIA RTX-2070 GPU with 8 GB memory. Both of them ran on converted two dimensional images from ECG signal by using various wavelet or Fourier transform.

### 3.1 Classification results using supervised machine learning models

In this study, two commonly used machine learning algorithms including KNN and SVM were used firstly to classify the R-TFM dataset, whose performances were used as a reference to further evaluate the CVAE based unsupervised model proposed in this work. As there two algorithms are supervised algorithms, the R-TFM of each pHFOs accompanying its label both are fed into the model, where all of real HFOs events including Rs and FRs are marked as a positive sample (1) whereas both Spikes and artifacts marked as a negative sample (0). Noted that the use of different kernel functions has an impact on the classification results of SVM.

As shown in Table 2, classification performances of the KNN model and the SVM model with three different kernel functions, such as linear kernel, polynomial kernel, and Gaussian kernel, are obtained in an average value in terms of the accuracy, sensitivity, and specificity, respectively. It can be seen that when classifying HFOs, the specificity of the KNN is higher than that of the SVM, while the sensitivity of the SVM is generally higher than that of the KNN. Moreover, the SVM model could achieve the optimal effect when using the Gaussian kernel function for classification.

**Table 2:** Classification results using supervised machine learning models.

Supervised Machine Learning Model	Accuracy (%)	Sensitivity (%)	Specificity (%)
KNN	89.87	86.22	93.64
SVM Linear kernel	89.61	93.60	86.24
SVM Polynomial kernel	88.93	90.39	85.58
SVM Gaussian kernel	90.08	93.67	86.27

### 3.2 Classification results using unsupervised machine learning models

In this study, four unsupervised models including mean-shift, K-means, GMM, and FCM were evaluated separately to perform HFOs cluster analysis on the R-TFM dataset. Table 3 shows the obtained performances of each unsupervised algorithm in terms of accuracy, sensitivity, and specificity, respectively.

**Table 3:** Classification results using unsupervised machine learning models.

Unsupervised Machine Learning Model	Accuracy (%)	Sensitivity (%)	Specificity (%)
Mean-shift	85.81	71.86	95.15
K-means	91.20	87.54	93.70
GMM	85.84	71.88	95.18
FCM	92.22	92.77	91.47

In comparison with supervised models as shown in the Table 2, all evaluated unsupervised models can generally achieve higher specificity as shown in Table 3, whereas a lower sensitivity could be observed in unsupervised models except the FCM one. Moreover, a comprehensive analysis of all indicators shows that FCM performs well in terms of accuracy, sensitivity, and specificity, with an average index of 92.15%. Consequently, compared with supervised algorithms as mentioned above, unsupervised algorithms do not need to input the labels of R-TFM datasets. Unsupervised algorithms can learn the characteristics of the data and cluster data with similar characteristics into a group. On the other hand, unsupervised algorithms measure across the entire dataset, so there is no need to train the model with cross-validation. As such, the FCM algorithm would be the optimal clustering model for HFOs detection.

### 3.3 Classification results using proposed CVAE model on reconstructed dataset

The proposed CVAE model was employed before cluster analysis so as to train the R-TFM dataset. Table 4 shows the obtained performance of HFOs classification using the proposed CVAE model together with four unsupervised algorithms described above on the reconstructed R-TFM dataset. It should be noted that 200 epochs were adopted as the maximum training epoch in this work so as to ensure the convergence of the network, because we found the loss of CVAE stopped decreasing after 200 epochs of training in our preliminary experiment, Compared to performances of an automated HFOs classification of both supervised and unsupervised models as shown Tables 2, 3, respectively, it can be found that all indicators of methods with the proposed CVAE model shown in Table 4 are superior in this work. Importantly, the FCM algorithm after CVAE training shows the highest performance among with four CVAE based clustering models, where the performances of in term of accuracy, sensitivity, and specificity are 93.02, 94.48, and 92.06%, respectively. It demonstrates clearly that that using the CVAE model for feature reconstruction combined with the unsupervised FCM clustering algorithm could achieve a superior performance of HFOs detection.

**Table 4:** Classification results using proposed CVAE models on reconstructed dataset.

Models	Accuracy (%)	Sensitivity (%)	Specificity (%)
CVAE + Mean-shift	91.05	85.51	94.64
CVAE + K-means	92.85	93.91	92.14
CVAE + GMM	86.78	75.08	90.89
CVAE + FCM	93.02	94.48	92.06

### 3.4 Classification results using FCM clustering on TFM, R-TFM, reconstructed R-TFM datasets

Additionally, one more evaluation of the unsupervised FCM clustering algorithm on various datasets was performed in this work including the TFM, the R-TFM, and the reconstructed R-TFM datasets, as shown in Table 5. We could find that there is an obvious improvement of classification performance of FCM clustering on the reconstructed T-TFM dataset in comparison to those on either the TFM or R-TFM datasets. Specifically, the obtained sensitivity of FCM clustering on the reconstructed R-TFM dataset is nearly 11% higher than that on the R-TFM dataset. In summary, the effectiveness of R-TFM reconstruction from original EEG data with the proposed CVAE model would be clear for an unsupervised FCM clustering to achieve a more accurate classification of HFOs in patients with epilepsy. Meanwhile, it should be noted that the classification performance of the unsupervised FCM model was found to be sensitive to the various latent layers and training epochs, as shown in Table 6. In this study, the R-TFM dataset was input into the CVAE for training. When the number of training epochs exceeded 200, the loss of the CVAE model stopped decreasing. Therefore, the maximum number of training epochs was set to 200 and a higher performance could be determined by using fifty latent layers of the unsupervised FCM model.

**Table 5:** Classification results of the unsupervised FCM model on various datasets.

Models	Datasets	Accuracy (%)	Sensitivity (%)	Specificity (%)
FCM	TFM	80.13	76.28	82.68
FCM	R-TFM	92.22	92.77	91.47
FCM	Reconstructed R-TFM	93.02	94.48	92.06

**Table 6:** Classification results of the unsupervised FCM model with various latent layers and training epochs.

latent layers *(l)*	epoch = 100			epoch = 150			epoch = 200		
	Acc (%)	Sen (%)	Sp (%)	Acc (%)	Sen (%)	Sp (%)	Acc (%)	Sen (%)	Sp (%)
10	90.03	93.44	89.05	93.39	94.41	92.72	92.58	94.35	91.40
20	91.13	93.91	91.24	92.26	93.79	91.24	92.45	94.29	91.24
30	91.87	92.99	93.25	92.87	94.29	91.94	92.62	94.23	91.57
40	90.65	94.00	91.94	92.13	93.73	91.07	92.53	94.58	91.20
50	91.19	93.74	89.56	92.40	93.67	91.57	93.02	94.48	92.06

## 4 Discussion

This study explores the application effect of a deep unsupervised algorithm in the automatic detection of HFOs signals and accordingly proposes an unsupervised clustering algorithm for HFOs detection based on CVAE model, which achieves a good performance in the automated detection of HFOs. Most of HFOs automatic detection algorithms that have been reported thus far are based on supervised learning. After obtaining the suspected HFOs signals from the EEG data, these methods still need to manually label them to assist them in training their machine learning model. This process is laborious, time-consuming, and prone to human interference. On the other hand, traditional unsupervised machine learning methods require the features of the input data as the basis for judgment. Therefore, researchers must analyze the HFOs signals before inputting the clustering model and perform feature extraction. The process of feature extraction is often vulnerable to prior knowledge. The impact leads to inaccurate feature extraction and affects the final model performance. Based on this, the algorithm proposed in this paper does not require label-assisted model training and automatically classifies data with similar characteristics in the database into one category. At the same time, the model combined with deep learning algorithms can automatically extract the characteristics of the input signal without manual operation, reducing the need for manual operations. The effect of prior knowledge of the process.

**Figure 4:**
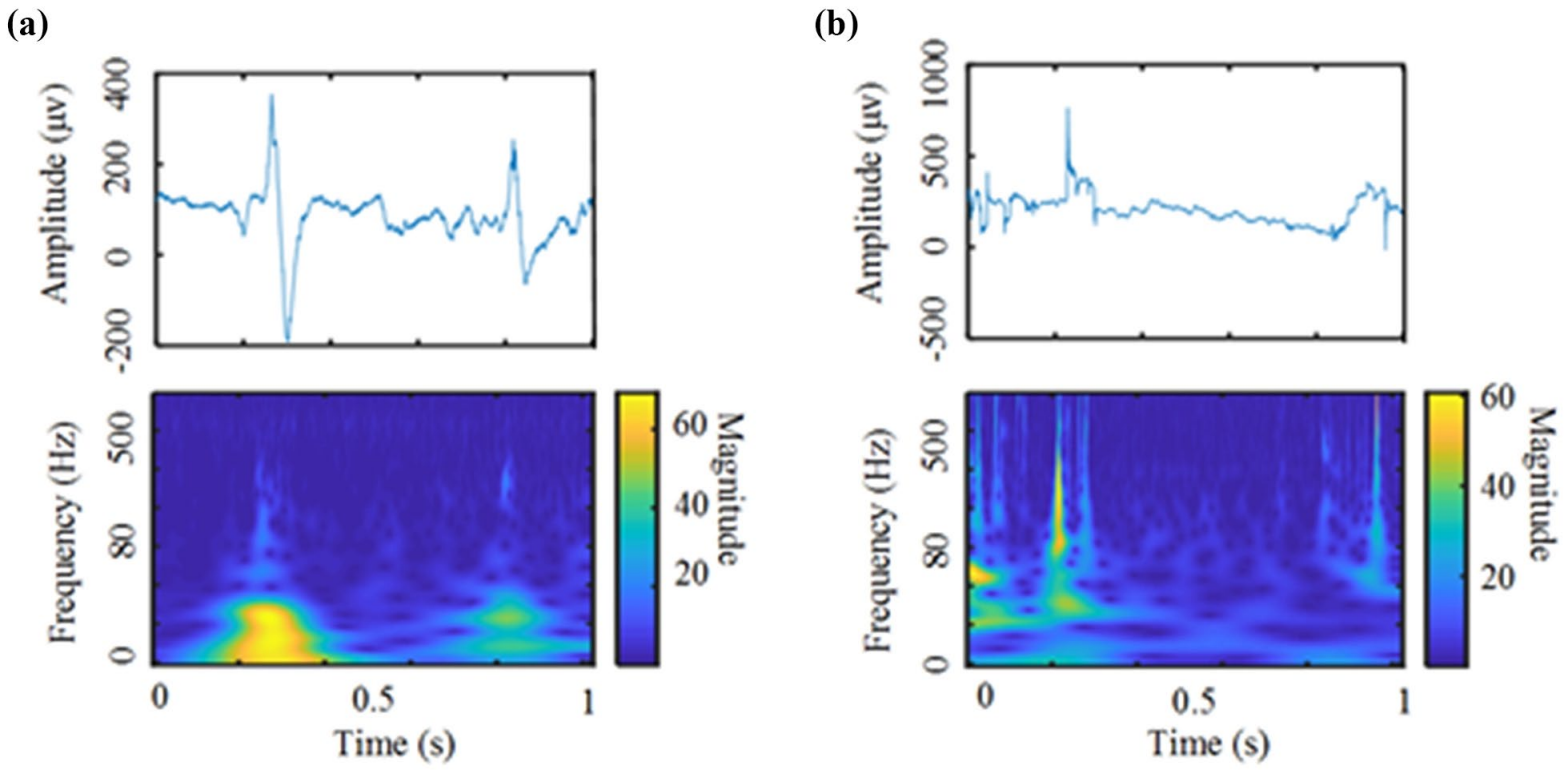
Two Examples of false HFOs: **(a)** a spike was detected as HFOs (Top: a spike without HFOs. Bottom: the time-frequency map of the spike). **(b)** A noise was detected as HFOs (Top: High-frequency noise signal. Bottom: the time-frequency map of the high-frequency noise).

For research on the automatic detection of HFOs, most researchers currently use supervised models, and fewer unsupervised models are used. This study explores the application of deep unsupervised models in the automatic detection of HFOs, and its performance even exceeds that of traditional supervised models. With unsupervised algorithms. Specifically, comparing Tables 2, 3, we find that when using the traditional unsupervised model to detect HFOs automatically, the performance indicators are close to those of the supervised model but still slightly inferior to those of the supervised model. Comparing Tables 4, 5, it can be found that we used the clustering model to analyze the dataset after CVAE training, and each performance index increased. Metrics are already surpassed by supervised algorithms. It should be noted that the presented study still has been suffering from false positives introduced by noise, epileptic spikes, and other oscillatory events that contain harmonics, which resulted in a decreased performance by several percent in either the sensitivity or the specificity, as shown in Table 5. Two Examples of false HFOs were shown in Figure 4, where a spike and a noise were both detected as HFOs. As such HFOs detection techniques have been widely discussed over recent 10 years as mentioned above, ongoing studies of the new biomarker of HFOs events would be meaningful for our currently limited understanding of the mechanism of epilepsy.

### 4.1 Comparison between previously reported and proposed methods

In the presented work, the motivation was to propose a novel unsupervised method using only two convolutional layers for encoders and decoders of the proposed CAVE model together with an initial detector of the STE detector to quickly distinguish HFOs segments from non HFOs such as spikes and artefacts, the structure of which is much simpler and straightforward than that of a sophisticated HFOs detection algorithm recently reported in (4) where the complex ResNet54 were used as the backbone for encoders and decoders. Moreover, the correspondingly obtained performance was therefore comprised those of recently reported supervised and unsupervised methods as cited references (5, 7, 19, 20, 28) as shown in Table 7, where all a binary classification of iEEG segments between of real HFOs and non HFOs was performed. Additionally, it should be noted that we mixed all pHFOs segments in one datasets and used a 5-fold cross validation rather than leave one subject in this work. The main reason why we used such cross-validation framework to evaluate the performance of our algorithm was of an engineering side so as to conduct an impartial comparison of performance reported by other studies, where a similar cross-validation framework was also employed as shown in references (5, 7, 19, 20, 28) in Table 7.

**Table 7:** Comparison of performance among of previously reported and proposed methods for automated detection of HFOs.

Reference	Methods	Accuracy (%)	Sensitivity (%)	Specificity (%)
Dümpelmann et al. (19)	Supervised Neural network	79.64	68.40	87.08
Liu et al. (5)	Unsupervised K-means	89.00	66.00	93.00
Jrad et al. (28)	Supervised SVM	85.06	83.80	85.89
Wu et al. (7)	Unsupervised EM-GMM based FCM	82.93	74.61	88.44
Ma et al. (20)	Supervised 2D-CNN	91.26	78.65	99.62
Proposed Study	Unsupervised CVAE based FCM	93.02	94.48	92.06

Moreover, we also found that a 2D-CNN model reported in (20) shows a relatively higher specificity with an obvious decrease in sensitivity compared with those of the proposed method. In conclusion, the comparison shows that our CVAE-based FCM clustering method provides a better performance of HFO classification in terms of accuracy than five existing detectors.

Specifically, According to references as described in Table 7. We reproduced five different algorithms for automatic HFOs detection and evaluated on the same clinical dataset collected in this work, including two supervised methods (19, 28) and two unsupervised methods (5, 7), and one more supervised deep learning algorithm using 2D-CNN (20). Compared with previous supervised machine learning models such as SVM and Neural networks reported in (19, 28). The method presented in this study does not require manual feature extraction and/or feature selection. Meanwhile, two traditional unsupervised algorithms including the K-means model (5) and the expectation–maximization-Gaussian mixture model (EM-GMM) were also reproduced in this work. However, their relatively lower performance in clustering HFOs would be an obstacle to their wide acceptance in comparison to the proposed method, which uses a novel deep neural network of the CVAE model.

### 4.2 Applications of the proposed study

Epilepsy is one of the most frequent chronic neurological diseases affecting an estimated number of 65 million people of worldwide and occurs in all age ranges (29). In clinical, pre-surgery, accurate localization of the epileptic foci in the patient being resistant to drug treatment is considered to be the key to successful surgical resection. However, identifying this brain region is challenging, as it is usually determined by trained clinicians through visual inspection of the massive intracranial electroencephalography (iEEG) data along with synchronized video, and there is no diagnostic modality available today to measure the epileptogenic zone directly. As HFOs in intracranial electroencephalograms have been proven recently to be a reliable biomarker of the epileptic foci, the concentrations of detected HFOs could be useful to localize epileptic zones in the pre-surgery iEEG signal analysis. With a deep CVAE model-based FCM clustering analysis in this work, the proposed method could automatically analyze iEEG signals in terms of a more informative representation of TFM and has the advantages of detecting HFOs and avoiding false detection caused by spikes and artifacts. By automatically detecting HFOs from the zone of seizure onset or at least estimating a region of interest containing or closed to the epileptogenic zone before the surgery procedure, the investigated unsupervised deep learning-based iEEG analysis technique would provide a useful and applicable pre-surgery guideline for the operator, and potentially reduce the time needed for long-term recording and manual inspection.

### 4.3 Limitations of the proposed study

This study also has some limitations. Due to the difficulty in collecting intracranial EEG signals, it is difficult for the patients in this study to cover multiple age groups and a variety of clinical symptoms. Therefore, it is necessary to continuously collect intracranial EEG data from epilepsy patients and expand patient data in our future work, and a larger and more diverse dataset with the leave-one-subject evaluation approach would strengthen our conclusions. Different clinical onset symptoms will be included to increase the generalization power of the model (30, 31). Meanwhile, further exploring subcategories of HFOs such as ripples (80–250 Hz) and fast ripples (250–500 Hz) (20, 32) as well as the use of recently reported explainable AI (XAI) techniques (33) in the unsupervised model would be meaningful, which could provide engineers with insights into how the model reconstructs the features and provide clinicians with insights into the underlying decision processes.

## 5 Conclusion

In this paper, we develop an automatic CVAE-based detector for unsupervised HFOs. For the first time, the deep learning models CVAE and 2D R-TFM are applied to HFOs detection. Extracting the red channel of the TFM can remove redundant information and capture salient features in the TFM. Furthermore, we use CVAE to perform automatic high-level feature extraction and reconstruction on the input features of the R-TFM dataset. Comprised of traditional supervised and other existing detectors for HFOs, a large number of comparative experiments are conducted to verify the effectiveness of the combination of the STE and CVAE. The superiority of our proposed detector with the best trained CVAE structure shows an advanced feature extraction as well as dimensionality reduction of EEG data by avoiding the need to handcraft or select features and manual annotation training manually, and thus would potentially serve to provide a clinically useful tool.

## Data Availability

The raw data supporting the conclusions of this article will be made available by the authors, without undue reservation.
